# Vitamin D deficiency and depression in obese adults: a comparative observational study

**DOI:** 10.1186/s12888-021-03586-4

**Published:** 2021-11-30

**Authors:** Leila Kamalzadeh, Malihe Saghafi, Seyede Salehe Mortazavi, Atefeh Ghanbari Jolfaei

**Affiliations:** 1 Mental Health Research Center, Psychosocial Health Research Institute, Department of Psychiatry, School of Medicine, Iran University of Medical Sciences, Tehran, Iran; 2 Minimally Invasive Surgery Research Center, Department of Psychiatry, School of Medicine, Iran University of Medical Sciences, Tehran, Iran; 3 School of Behavioral Sciences and Mental Health (Tehran Institute of Psychiatry), Iran University of Medical Sciences, Tehran, Iran

**Keywords:** Vitamin D deficiency, 25-hydroxyvitamin D, Obesity, BMI, Depression

## Abstract

**Background:**

Amongst the contributing factors of depression, vitamin D deficiency has increasingly drawn attention in recent years. This paper seeks to examine the association between serum vitamin D level and depression in patients with obesity.

**Methods:**

In this comparative observational study, serum 25-hydroxyvitamin D [25(OH)D] levels were compared between obese individuals with depression (*n* = 174) and those without depression considering the effect of potential confounders. Participants were selected from males and females aged 18 to 60 years old visiting the outpatient obesity clinic of Rasoul-e Akram hospital, Tehran, Iran. The diagnosis of depressive disorder was made based on the Diagnostic and Statistical Manual of Mental Disorders (DSM-5) criteria. Additional clinical and laboratory data were collected from hospital electronic records. Mann–Whitney U test (nonparametric), Student’s t-test (parametric), and Chi-squared test were used to analyze the differences between the two groups. To examine age and gender differences in the relationship between vitamin D deficiency and depression, stratified analyses were conducted by age and gender groups.

**Results:**

The mean 25(OH) D levels were significantly different between depressed and non-depressed groups (20 ± 15 vs. 27 ± 13, *P* <  0.001). Vitamin D insufficiency/deficiency was detected in 78 and 67% of the depressed and non-depressed groups, respectively, which was significantly different (*P* = 0.03). The associations between depression and the serum 25(OH) D levels were observed regardless of gender and age. The overall average vitamin D levels were not significantly different between total males and females (22 ± 13 vs. 23 ± 14, *P* = 0.49). The average level of vitamin D was higher in the older age group (40–60 years) compared to younger participants (18–39 years) (26 ± 15 vs. 21 ± 13, *P* = 0.004).

**Conclusion:**

The present study provides additional evidence for the hypothesis that low vitamin D serum concentration is associated with depression in obese adults, and highlights the need for further research to determine whether this association is causal.

## Introduction

Obesity is increasingly recognized as a serious public health concern. More than 650 million people throughout the world suffer from overweight and obesity [1]. There is strong evidence to suggest that obesity is conjoint with several mental and physical problems [2]. Depression is one of the most prevalent psychiatric disorders reported in the obese population contributing to significant disability, mortality, and healthcare costs [3]. The exact mechanisms linking depression and obesity have not been established. Studies have shown multiplex interactions between biological, psychological, and environmental factors giving rise to the association between obesity and depression [4]. Amongst the contributing factors of depression, vitamin D deficiency has increasingly drawn attention in recent years [5].

Vitamin D, also known as cholecalciferol, is a unique neuro-steroid hormone that is vital for numerous brain functions. This hormone binds to receptors in numerous brain regions including the hippocampus and cingulate cortex, which are involved in the pathogenesis of depression and other mental illnesses [6]. Many clinical studies have found depression, anxiety and cognitive impairment to be associated with low serum levels of 25-hydroxyvitamin D [25(OH)D], which is the major circulating form of vitamin D, in average weight people [5–7]. Recent research has also provided evidence for antidepressant properties of vitamin D supplementation [8, 9].

Strong evidence of aberrations in the vitamin D-endocrine system as well as low serum 25(OH) D levels have been seen in obese individuals [10, 11]. It has been demonstrated that vitamin D deficiency is 35% more likely in obese people compared to healthy-weight subjects [12]. Volumetric dilution of Vitamin D is the most plausible mechanism of low 25(OH) D in individuals with obesity [11]. Alternative mechanisms for lower 25(OH) D concentrations in patients with obesity include lower dietary consumption, decreased dermal synthesis, reduced intestinal absorption, altered metabolism, as well as less sunlight exposure due to lower physical activity [13].

Despite a biologically potential role of vitamin D in the development of depression, very few studies have investigated this association in overweight and obese subjects, with conflicting findings. While two studies indicated the benefits of vitamin D supplementation on depressive symptoms in obese adults [14, 15], a recent randomized trial presented contradictory results [16].

The discrepancy among these results has given rise to the need for further research. In this regard, this study seeks to examine the association between serum vitamin D level and depression in obese patients.

## Methods

### Study design

This comparative observational study was conducted at Rasoul-e Akram hospital, an Iran University of Medical Sciences affiliate located in Tehran, Iran.

### Study participants

The study population consisted of males and females aged 18 to 60 years old visiting out-patient obesity clinic from April 2019 to October 2020. The participants were selected using convenience sampling. Based on the presence or absence of depressive symptoms, participants were categorized into two separate groups. The diagnosis of depressive disorder was made by expert psychiatrists using the Structured Clinical Interview for the Diagnostic and Statistical Manual of Mental Disorders (DSM-5) (SCID-5) [17]. All participants had documented serum 25-hydroxyvitamin D, thyroid-stimulating hormone (TSH), fasting blood sugar (FBS), and parathyroid hormone (PTH) levels from their screening laboratory tests performed in the obesity clinic within 2 weeks before study enrollment. Additional clinical, anthropometric, and demographic data were collected from hospital electronic records. Eligibility criteria required patients to have a body mass index (BMI), calculated as weight/height^2^ (kg/m^2^), greater than or equal to 30 kg/m^2^, normal TSH, normal FBS, normal PTH level, no history of sleep apnea, neither past nor present substance use, no history of calcium supplement or vitamin D use for at least 1 month before study enrollment. Additional criteria for the non-depressed group included: not meeting criteria for another mental disorder and no history of psychiatric medication use. Patients with recent illnesses or incomplete medical records were excluded from the study.

### Measurements

All laboratory evaluations were performed in the morning after an overnight fast using reliable assays. The serum 25(OH) D measurements were classified according to definitions established by the Endocrine Society: 30–100 ng/ml was considered normal, with insufficient 25(OH) D levels or hypovitaminosis D sub-grouped into two: vitamin D insufficiency (20–29 ng/ml) and vitamin D deficiency (< 20 ng/ml) [18]. Considered laboratory reference ranges for other biochemical parameters were: TSH (0.35–5.50 mIU/L), FBS (< 100 mg/dl), and PTH (0–55 pg/mL).

### Statistical analysis

Categorical data were expressed as counts and percentages. Continuous variables were presented as mean and standard deviation (SD). The Statistical Package for Social Sciences, version 22 was used for all statistical analyses (SPSS Inc., Chicago, IL, USA). The normal distribution of the variables was examined using Kolmogorov- Smirnov test. Mann–Whitney U test (nonparametric), Student’s t-test (parametric), and Chi-squared test were used to analyze the differences between two groups. To examine age and gender differences in the relationship between vitamin D deficiency and depression, stratified analyses were conducted by age and gender groups. A *p*-value of < 0.05 was considered statistically significant.

## Results

A total of 174 depressed patients and 173 non-depressed individuals were studied. The baseline characteristics of the participants are summarized in Table 1. Statistically significant differences were observed among the two groups in terms of the ratio of females to males (*P* <  0.001) and age (P <  0 .001), i.e., the depressed group had a significantly higher proportion of females (92% vs. 75%) and a higher mean age than the non-depressed group (42 ± 10 vs. 37 ± 10). Moreover, vitamin D insufficiency/deficiency was detected in 77 and 67% of the depressed and non-depressed groups, respectively, which was significantly different (*P* = 0.03). The mean BMI was not significantly different between the groups (*P* = 0.05). The mean vitamin D levels were not significantly different between males and females (22 ± 13 vs 23 ± 14, *P* = 0.49).

**Table 1:** Baseline characteristics of the participants

Characteristic	Depressed *N* = 174	Non-depressed *N* = 173	*P* value
Age (years)	42 ± 10	37 ± 10	< 0.001
18–39 years, n (%)	72 (40%)	111 (60%)	
40–60 years, n (%)	102 (62%)	62 (38%)	
Female, n (%)	160 (92%)	130 (75%)	< 0.001
BMI (Kg/m^2^)	42 ± 7	43 ± 7	0.05
Vitamin D status, n (%)			
Normal	39 (22%)	56 (32%)	0.03
Insufficient/Deficient	135 (77%)	117 (67%)	

As can be seen from Table 2, the average vitamin D levels were significantly different between the depressed and non-depressed groups (20 ± 15 vs. 27 ± 13, *P* <  0.001). Moreover, the results obtained from separate analyses on variable levels of gender, showed that the mean vitamin D level for the depressed men was significantly lower than non-depressed men (17 ± 14 vs. 24 ± 12, *P* = 0.01). Similarly, depressed women had a notably lower mean vitamin D level compared to non-depressed women (21 ± 16 vs. 27 ± 13, P <  0.001). Therefore, the association between depression and the serum 25(OH) D levels was observed regardless of gender.

**Table 2:** Relationship between depression and the serum 25-hydroxyvitamin D levels totally and by Gender and Age

	Non-Depressed		Depressed		*P*-value
	Vitamin D		Vitamin D		
	Mean	Standard Deviation	Mean	Standard Deviation	
Total	27	13	20	15	< 0.001
Gender					
Male	24	12	17	14	0.01
Female	27	13	21	16	< 0.001
Age					
18–39	25	12	16	13	< 0.001
40–60	29	14	23	16	0.03

As shown in Table 2, the mean serum vitamin D levels appear to be generally lower in men participating in this study compared to women. Nonetheless, these differences were not statistically significant. In other words, the mean vitamin D level in depressed men did not differ significantly from depressed women (17 ± 14 vs. 21 ± 16, *P* = 0.28). Likewise, the mean vitamin D level in non-depressed men did not differ significantly from non-depressed women (24 ± 12 vs. 27 ± 13, *P* = 0.10). The overall average vitamin D levels were also not significantly different between total males and females (22 ± 13 vs 23 ± 14, *P* = 0.49).

Regarding age groups, our finding revealed that the mean vitamin D level for the depressed participants aged 18–39 years was significantly lower than that of non-depressed aged 18–39 years (16 ± 13 vs. 25 ± 12, *P* < 0.001). In the same way, depressed participants aged 40–60 years had a notably lower mean vitamin D level compared to non-depressed aged 40–60 (23 ± 16 vs. 29 ± 14, *P* = 0.03). Therefore, the association between depression and the serum 25(OH) D levels was observed regardless of age. The overall average vitamin D levels were also significantly different between age groups 18–39 and 40–60 (21 ± 13 vs 26 ± 15, *P* = 0.004).

## Discussion

The findings of the present study revealed that there is an association between low serum 25(OH) D levels and incident depression in obese patients. The relation between low levels of vitamin D and depression was already established in normal-weight individuals by past research [5–7]. However, relatively few studies have evaluated this relationship in obese adults, and results have been mixed. Consistent with our findings Jorde et al. [15] and Milaneschi et al. [4] found that a low serum level of vitamin D is a risk factor for depression and suggested that BMI plays an important mediating role in the association between vitamin D and depression. In the same vein, Irandoust et al. [14] demonstrated that both vitamin D supplementation and physical activity have a beneficial impact on depressive symptoms in obese females. Penckofer et al. [19] yielded comparable results in their study on women with type 2 diabetes. They demonstrated that vitamin D supplementation significantly ameliorated depression in the patients.

Multiple mechanisms are involved in the interaction between vitamin D deficiency, obesity, and depression. It has been proposed that an inverse relationship exists between vitamin D serum levels and BMI [20]. As was pointed out in the introduction to this paper, low serum 25(OH) D in obese patients can occur for many reasons including insufficient vitamin D consumption, increased fat or muscle mass, genotype variation in vitamin D binding proteins or enzymes responsible for vitamin D metabolism [11, 13]. On the other hand, vitamin D deficiency can increase the risk of developing depression, through several biological pathways including effects on immunomodulation, regulation of intracellular calcium stores, cellular signaling, modulation of the hypothalamic-pituitary-adrenal axis, intracellular calcium homeostasis, and production of neurotransmitters [6, 21]. Moreover, both obesity and vitamin D deficiency lead to chronic low-grade inflammation, which has been suggested to contribute to the development of depression [11, 22]. At last, it is also possible that PTH levels contribute to the relation between vitamin D, BMI, and depression. Recent evidence suggests that both obesity and low vitamin D levels are accompanied by significantly higher PTH levels, and high PTH levels are related to depression [4].

Indeed, the possibility of reverse causality should also be noted. In other words, certain features associated with depression and obesity might have limited the vitamin D sources and increased the risk of vitamin D deficiency in the depressed participants of this study, as compared with the non-depressed group. For example, depressed individuals with obesity may avoid outdoor activity and sunlight exposure for long periods due to body image concerns, social withdrawal, anhedonia, particular personality traits, and pathological fatigue [6, 23]. Furthermore, poor quality diet and metabolic derangements associated with obesity and depression can increase the demand for vitamin D (for restoring calcium homeostasis) and heighten the risk of vitamin D deficiency [24].

However, more recently, literature has emerged that offers contradictory findings on these associations. In contrast to the mentioned studies, in a randomized controlled study, Mousa et al. [16] showed that depressive symptoms in obese individuals were not associated with 25(OH) D concentrations, nor did improve by Vit D supplementation. Similarly, a recent clinical interventional cohort study revealed that vitamin D supplementation for 6 months had no significant impact on depressive symptoms, but could improve anxiety symptoms in depressed patients with vitamin D deficiency. The authors suggested that BMI is an important mediating factor between low serum 25(OH) D and anxiety symptoms [25].

Different findings may be attributed to the differences in the study population (different races, gender, and age groups), diverse methodology (different vitamin D supplementation doses and duration), and different baseline levels of serum 25(OH) D concentrations. Moreover, the mentioned studies applied self-reported psychiatric rating scales for evaluation of depression rather than a clinician-rated assessment and therefore were prone to multiple potential biases. In addition, some of the previous studies did not adjust for potential confounders, such as thyroid dysfunction, comorbid diabetes mellitus, sleep apnea, low physical activity, and substance use that may play a role in developing depression in the obese population [26].

As mentioned before, in this study depressed patients had significantly lower mean vitamin D levels than the non-depressed group; however, the distribution of gender groups in our study was quite imbalanced (overrepresentation of women). Considering biological and psychosocial differences between males and females, we also examined the relationship between vitamin D status and depression in men and women separately. In both genders, the mean vitamin D levels were significantly lower in the depressed group compared to nondepressed individuals. The finding is in the lines of earlier literature that found no significant interaction effect of gender on the association between vitamin D status and depression [6, 27]. Furthermore, in this study, the average vitamin D levels were not significantly different between male and female participants; however, it ought to be remarked that among the morbidly obese females, the risk of developing depression related to vitamin D deficiency could be higher than males owing to some psychosocial variables such as stigmatization, discrimination, poor self-esteem, social expectations, and gender-role attitudes resulting in further reduction of natural sources of vitamin D [28–30]. The results further demonstrated that vitamin D deficiency was associated with depression in both age groups. This association appeared to be even more robust in younger participants. A similar pattern of results has been obtained in other studies investigating the associations between vitamin D levels and depression across different age groups [6, 31, 32]. One unanticipated finding was that the average level of vitamin D was higher in the older age group compared to younger participants. Previous research has suggested that aging may contribute to vitamin D deficiency through several mechanisms including reduced renal production of 1,25(OH)_2_D, decreased skin production of vitamin D, and substrate deficiency of vitamin D [33]. It seems possible that our results are due to dietary supplement use which is common in older adults in Iran [32].

Several limitations to this study need to be acknowledged. First, the sample size was relatively small. Therefore, larger studies will be needed to confirm our observations. Second, the depressed and non-depressed groups were not matched based on age, gender, and season of collecting vitamin D. Recent evidence suggests that the circulating levels of 25(OH) D can be affected by acute illness, diurnal rhythm, and season of blood draw [34, 35]. However, it is noteworthy that participants were selected from the same hospital catchment area, and therefore had similar socioeconomic status. The key strength of this study is the exclusion of several potential confounders such as thyroid dysfunction, comorbid diabetes, hyperparathyroidism, and substance use which were not addressed by previous studies. Moreover, in the present study, the diagnosis of depression was based on standardized semi-structured interviews by expert psychiatrists rather than self-report rating scales.

## Conclusions

In summary, the present study provides additional evidence for the hypothesis that low vitamin D serum concentration is associated with depression in obese adults, and highlights the need for further research to find out whether this association is causal.

## Data Availability

The datasets used and/or analyzed during the current study are available from the corresponding author on reasonable request.

## Abbreviations

BMI: Body Mass Index
DSM-5: Diagnostic and Statistical Manual of Mental Disorders
EIA: Enzyme Immunoassay
FBS: Fasting Blood Sugar
25(OH)D: 25-Hydroxyvitamin D
PTH: Parathyroid Hormone
SCID-5: Structured Clinical Interview for DSM-5
SPSS: Statistical Package for Social Sciences
TSH: Thyroid-Stimulating Hormone
